# The Shortcomings of COVID-19 Testing in Ecuador: Time to Incentivize Research and Innovation

**DOI:** 10.3390/life12030325

**Published:** 2022-02-22

**Authors:** Izan Chalen, María Mercedes Cobo, Bernardo Gutierrez, Andrés Carrazco-Montalvo, Patricio Ponce, Diego F. Cisneros-Heredia

**Affiliations:** 1 Colegio de Ciencias Biológicas y Ambientales COCIBA, Universidad San Francisco de Quito USFQ, Quito 170901, Ecuador; mcobo@usfq.edu.ec (M.M.C.); bgutierrezg@usfq.edu.ec (B.G.); andres.carrazco@hotmail.com (A.C.-M.); 2 Beckman Institute, University of Illinois Urbana-Champaign, Urbana, IL 61801, USA; 3 Instituto Ibiotrop, Universidad San Francisco de Quito USFQ, Quito 170901, Ecuador; 4 Department of Paediatrics, University of Oxford, Oxford OX3 9DU, UK; 5 Department of Zoology, University of Oxford, Oxford OX1 3SZ, UK; 6 Instituto Nacional de Investigación en Salud Pública, Quito 170136, Ecuador; wponce@inspi.gob.ec

**Keywords:** coronavirus, research and development, diagnosis, biotechnology industry

## Abstract

The COVID-19 pandemic hit Ecuador severely. The country caught the attention of international media due to its high death toll and overwhelmed healthcare system. The clinical diagnostics system was rapidly overloaded, and the import of PCR tests was delayed. The case of Ecuador illustrates how middle-income countries rely heavily on the importation of biotechnological products for their healthcare systems. The Ecuadorian experience during the COVID-19 pandemic serves as a call for the formation of policies for the development of the biotechnological industry.

Ecuador has been one of the hardest-hit Latin American countries during the COVID-19 pandemic [1]. The initial ballooning of cases in Guayaquil during the first weeks of the epidemic received international media attention due to the high death toll and the overwhelmed healthcare system [2]. Nationwide, the number of confirmed COVID-19 deaths per million people is high compared to other countries—but as these numbers have accumulated over time, the daily number of diagnostic tests has not scaled accordingly (Figure 1A). Numerous countries with developing economies have faced challenges establishing effective mass-testing strategies [3], and these tend to be countries with underdeveloped healthcare/biotechnology industries (Figure 1B). The experience from countries with solid testing regimes shows that widespread testing is an essential tool for identifying and containing pockets of transmission promptly [3] and for supporting surveillance efforts [4].

In March 2020, during the first week of the epidemic in Ecuador, the National Institute for Public Health Research (INSPI) received 6826 samples for COVID-19 diagnosis using RT-PCR in its main lab in the city of Guayaquil. However, the maximum testing capacity of the INSPI laboratory in Guayaquil—the only one in the country authorized to process those samples at the time—was 350 tests per day. By March 18th, the INSPI laboratories in Quito and Cuenca had also begun to process samples to assist with the high testing demand. However, at this point, the entire diagnostics system was quickly overwhelmed, so much so that the delayed sample processing would not have been brought up to date until the end of April 2020 [5]. At this time, several private universities and specialized diagnostics laboratories began processing samples, some with a cost of up to $120 per person. Notably, the limiting factor for diagnostic scaling was the lack of PCR testing kits rather than the unavailability of certified laboratories. The often inaccessible cost of taking a test in the private sector further deepened the testing problems in the country, an issue that was also exacerbated by severe delays in importation times of essential consumables and reagents.

The diagnostic gold standard for COVID-19 is the polymerase chain reaction (PCR), which is a fundamental technique in diagnosing ongoing SARS-CoV-2 infection. The availability of equipment and reagents needed for real-time PCR diagnostics in many low- and middle-income countries depends entirely on importations, which can be lengthy, bureaucratic, and costly. Importation issues ultimately delay the implementation of widespread testing, increasing the turnover times of test results. In Ecuador, private businesses can require up to 7 weeks for their imports to arrive. This may take several months in the public sector due to the mandatory public procurement procedures and complex processes for selecting qualified local suppliers. 

The financial burden of this reliance on importations is considerable, with final supply costs reaching as high as 45% above the original retail price. The Ecuadorian government has granted tariff relief to institutions of higher education, yet universities can still only reduce their importation costs to 25% above the actual retail price. Moreover, the successful application of this tariff reduction scheme is slow—it can take at least 12 weeks for supplies to arrive.

The application processes that public institutions need to go through to acquire and import tax-free goods are complex and time-consuming under normal conditions, let alone under the high global demand for COVID-19 testing reagents when expediency is essential in securing key assets to maintain a national surveillance program. These challenges call for local and regional capacity building to counteract the negative effect of productive and technological dependency for key reagents and supplies. Despite these challenges, tariff benefits have allowed several universities to provide critical support to testing programs in the country.

During the pandemic, academia has become a significant ally to the health sector by providing expertise and data analysis [6], building up complementary testing capacity, developing clinical solutions [7], and performing genomic surveillance [8]. Ecuadorian law exempts higher education institutions from paying importation tariffs for goods to be used in research or teaching. However, it was unclear whether these efforts pertained to academic research, posing an additional challenge to establish whether these tributary exemptions were extended to resources destined to provide health services, such as diagnostics. Universities with more robust institutional structures were able to address these bureaucratic, legal, and financial challenges earlier in the health emergency, resulting in a predominant role from academic institutions in cities such as Quito (Ecuador’s capital) to support the response to the pandemic. Institutions in other regions of the country were delayed or unable to support testing programs or actively engage in research related to COVID-19. 

Unfortunately, the limitations mentioned above are not new for Ecuadorian scientists. Before the beginning of the COVID-19 pandemic, the elevated costs of laboratory supplies and the delays during importation processes were already a burden for academic research [4]. The current health emergency has brought home the urgency of addressing these challenges by improving the conditions in which research is performed and promoting the development of the local biotechnology industry for the medical and healthcare sectors to better handle current and future health challenges in the country [9]. 

Ecuador, over the course of the last decade, has made progress amassing highly skilled and specialized human resources, increasing its ability to develop innovative biotechnological solutions which would benefit from competitive incentive programs. The COVID-19 pandemic has shown how nations can generate inventive designs for facing mass diagnostic challenges [10]; however, performing laboratory-based diagnosis is impossible without the resources that the biotechnology industry provides [9]. This is particularly important given that SARS-CoV-2 features a remarkable adaptive capacity derived from its high evolutionary rate and transmissibility, highlighting the need for diagnostic capabilities that can match the dynamic nature of the pandemic [11,12,13]. Furthermore, the emergence of new variants such as Omicron (detected in Ecuador in December 2021 [14]) emphasizes the value of incorporating genomic surveillance to the diagnostic pipelines to identify the drivers of viral evolution and to respond with appropriate public policies that can be translated from international scenarios to national and local realities [11,14,15]. 

The Ecuadorian experience during the COVID-19 pandemic illustrates critical shortcomings that other middle-income countries may be experiencing and serves as a call for the establishment of strategies for biotechnological development. An area to be prioritized is the development of products for genomic surveillance and clinical diagnostics, including but not limited to the synthesis of oligonucleotides and the production of antibodies. Such strategies will require participation and contributions from multiple fronts including the public and private sectors, industry, and academia and the establishment of favorable social and commercial conditions for research and innovation.

## Figures and Tables

**Figure 1:**
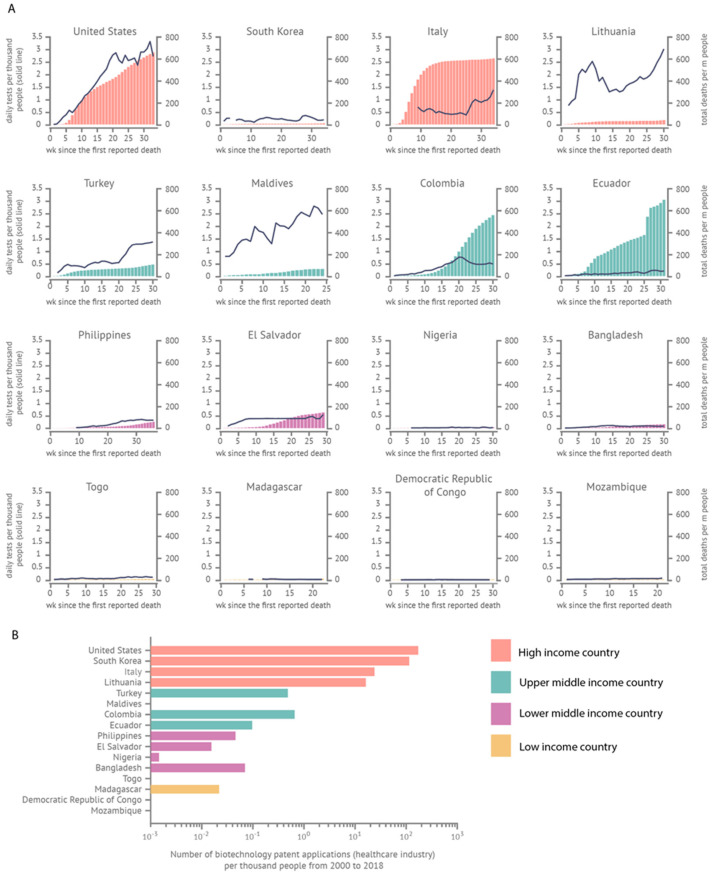
Impact of the COVID-19 pandemic, testing, and biotechnology industry in different countries. (**A**) Comparison of daily tests per thousand people and total deaths per million people in selected countries—high- (United States, South Korea, Italy, and Lithuania), upper-middle- (Turkey, Maldives, Colombia, and Ecuador), lower-middle- (Philippines, El Salvador, Nigeria, and Bangladesh), and low-income countries (Togo, Madagascar, Democratic Republic of Congo, and Mozambique)—during the first 30 weeks since the first reported death. (**B**) Number of biotechnological patent applications (healthcare industry) per thousand people from 2000 to 2018 in selected countries—high- (United States, South Korea, Italy, and Lithuania), upper-middle- (Turkey, Maldives, Colombia, and Ecuador), lower-middle- (Philippines, El Salvador, Nigeria, and Bangladesh), and low-income countries (Togo, Madagascar, Democratic Republic of Congo, and Mozambique).

## Data Availability

The data that support the findings of this study are openly available in Our World in Data https://ourworldindata.org/coronavirus (accessed on 10 January 2022) and PATSTAT v.2.6.8 from the European Patent Office https://data.epo.org/expert-services/index.html (accessed on 10 January 2022).
